# Genetic architecture of threshold reaction norms for male alternative reproductive tactics in Atlantic salmon (*Salmo salar* L.)

**DOI:** 10.1038/srep43552

**Published:** 2017-03-10

**Authors:** Olivier Lepais, Aurélie Manicki, Stéphane Glise, Mathieu Buoro, Agnès Bardonnet

**Affiliations:** 1 ECOBIOP, INRA, Univ. Pau & Pays Adour, 64310, Saint-Pée-Sur-Nivelle, France

## Abstract

Alternative mating tactics have important ecological and evolutionary implications and are determined by complex interactions between environmental and genetic factors. Here, we study the genetic effect and architecture of the variability in reproductive tactics among Atlantic salmon males which can either mature sexually early in life in freshwater or more commonly only after completing a migration at sea. We applied the latent environmental threshold model (LETM), which provides a conceptual framework linking individual status to a threshold controlling the decision to develop alternative traits, in an innovative experimental design using a semi-natural river which allowed for ecologically relevant phenotypic expression. Early male parr maturation rates varied greatly across families (10 to 93%) which translated into 90% [64–100%] of the phenotypic variation explained by genetic variation. Three significant QTLs were found for the maturation status, however only one collocated with a highly significant QTL explaining 20.6% of the variability of the maturation threshold located on chromosome 25 and encompassing a locus previously shown to be linked to sea age at maturity in anadromous Atlantic salmon. These results provide new empirical illustration of the relevance of the LETM for a better understanding of alternative mating tactics evolution in natural populations.

Alternative mating tactics found in natural populations of diverse species have important ecological and evolutionary implications. At the individual level, different mating tactics often involve variation in life history traits such as time to maturation, habitat use and reproductive behaviour^1^,^2^. This may in turn affect life cycle characteristics such as mating systems, generation time and reproductive structure which will impact effective population size and population dynamics^3^. Therefore, factors influencing the occurrence of alternative mating tactics have been the focus of longstanding theoretical and empirical research to decipher the relative contribution of genetic, environmental effects^1^,^4^,^5^ and their interactions through phenotypic plasticity^6^,^7^.

One of the most recent conceptual representation of alternative mating tactics is the environmental threshold model, which links the environment to alternative phenotypes through a genetically-determined threshold^7^,^8^,^9^. This model derives from the quantitative genetics threshold model^10^ and involves two components: (i) a normally distributed trait (liability trait) under polygenic and environmental influences integrating, for instance, individual physiological responses to the environment that can be assessed by a surrogate measure such as body size or weight; and (ii) a genetically determined threshold that will determine the decision to develop one particular life history trait. According to this model, incidence of alternative phenotypes can evolve through genetic change of the threshold value in addition to multigenic or environmental change of the distribution of the liability trait reflecting phenotypic plasticity^6^. As this model explicitly predicts that alternative phenotypes can be expressed by a given genotype depending on the environment, it provides a useful conceptual framework to study phenotypic plasticity^7^,^11^. Yet, in spite of formal recent statistical developments that should facilitate its confrontation to experimental data^6^, empirical application of this model remains rare^12^,^13^,^14^,^15^. Furthermore, most available measurements were obtained under rearing conditions which may lead to spurious results. Additional insight into the genetic architecture and phenotypic plasticity of alternative mating tactics should be gained in more ecologically realistic settings to further our understanding of the evolutionary processes maintaining alternative mating tactics in natural populations^16^. The objective of the present study is to apply the environmental threshold model in an ecologically-relevant context to better understand the genetic basis and the genetic architecture of the components of alternative reproductive tactics.

Salmonids species are a preferred model for ecological genetics studies^17^. Among them, we chose the Atlantic salmon (*Salmo salar* L.), a suitable model for the study of the evolution of alternative mating tactics in vertebrates given extreme variability in reproductive tactics observed among males^18^. Indeed, while the species is described as anadromous, i.e. individuals arising in freshwater only mature after completing a marine migration before returning to freshwater to spawn, some young males mature early before their seaward migration (mature parr, Fig. 1a). Mature male parr take advantage of their small size that can be up to two or three order of magnitude smaller than anadromous males (Fig. 1a) to sneak into female nests and furtively gain paternity^19^. This alternative mating tactic probably evolved among males to escape strong competition for access to females^18^. Prevalence of male parr maturation is highly variable between rivers^20^. While mature male parr reproductive contribution can exceed anadromous male reproductive contribution at the population level (e.g. 25 to 56%^19^ and up to 87%^21^ of male reproductions in the Nivelle population in south of France), mature male parr individual reproductive success is far lower than anadromous male individual reproductive success due to a reduced matting success of mature male parr^19^. Evidence from experimental studies including artificial crosses followed by rearing in laboratory conditions and from studies in natural populations has demonstrated a genetic component for early male maturation in *S. salar*^15^,^22^,^23^,^24^,^25^,^26^,^27^. However, the absence of genetic effects on early male maturation has also been reported in a natural population based study^28^ showing instead epigenetic effects suggesting a role for gene expression regulation inheritance. Nevertheless, environmental factors play an important role in determining early maturation decision, calling into question the relevance of results obtained in laboratory conditions^22^,^24^,^29^,^30^. Indeed, laboratory conditions are not representative of those experienced in natural populations (particularly when individuals are fed with commercial pellets optimised for growth) and may induce selective mortality and bias natural phenotypic expression. Here, we took advantage of unique experimental facilities to rear progeny derived from artificial crosses in a semi-natural channel allowing the expression of natural fish development and behaviour to be preserved. Furthermore, alternative mating tactics depend on complex genetic and environmental interactions which need to be dissected to tease apart environmental effects from heritable component to better understand evolutionary constrains of such puzzling life history trait. In this paper, we first describe genetic and environmental effects on weight-at-age (as a proxy of growth rate) and incidence of early maturation of juvenile male of the year. Then we investigate the relationship between early growth rate and incidence of early male maturation by applying the conceptual framework of the environmental threshold model to estimate maturation threshold distribution and heritability and quantify maturation reaction norms, i.e. the relationship between early growth rate and incidence of early male maturation^11^, within family and rearing environment. Finally, we explore for the first time the genomic aspect of the different components of early male parr maturation by performing QTL analyses of early growth, maturation status and threshold to characterize the genetic architecture of alternative reproductive tactics.

## Results

### Fish rearing

From a total of 1200 *S. salar* alevins released at the start of the experiment, 755 juveniles were caught in spring (407 upstream and 348 downstream of the experimental channel) and 735 at the end of the experiment (411 upstream, 324 downstream). After genotyping, genotype detected only once (spring n = 80, fall n = 81) were removed from subsequent analysis. Therefore, subsequent analyses were conducted on 344 and 300 individuals that were fished twice upstream and downstream respectively, from which full biometrical data (sex, spring weight and maturity) were recorded. Parentage assignment gave unambiguous identification of 75 to 106 individuals per family upstream and 70 to 84 individuals per family downstream (Fig. 2). Within each reach, survival in October was not significantly different across families (upstream section: *χ*^2^ = 3.39, d.f. = 3, *p* = 0.33; downstream section: *χ*^2^ = 1.57, d.f. = 3, *p* = 0.66). Sex ratio was balanced for each family and channel reach (Fig. 2, all chi-squared test not significant).

### Early growth rate

Offspring sex set as a main factor (*F*_1, 632_ = 0.05, *p* = 0.821) or in interaction with cross (*F*_3, 632_ = 0.29, *p* = 0.83) or habitat (*F*_1, 632_ = 2.17, *p* = 0.14) did not explain differences in offspring spring weight. On the contrary, cross (*F*_3, 632_ = 33.15, *p* < 0.001) and habitat (*F*_1, 632_ = 98.00, *p* < 0.001) as main effects had a significant impact on offspring spring weight, but not their interaction (*F*_3, 632_ = 1.44, *p* = 0.23). Spring weight of male individuals varied significantly between crosses (*F*_3, 334_ = 18.37, *p* < 0.001) and habitats (*F*_1, 334_ = 72.14, *p* < 0.001) with a marginally significant interaction effect (*F*_3, 334_ = 2.40, *p* = 0.07). Multiple comparisons of the mean (Fig. 3) indicate a significantly faster growth in the downstream reach (mean and 95% confidence interval difference: 0.93 [0.71–1.14] g, *p* < 0.001) and a family effect with offspring from F3xMP3 family growing faster than the other families (difference: ranging from 0.44 to 0.82 g, all comparisons *p* < 0.001). Within family and reach, there was generally no consistent pattern of differences in growth for male offspring fathered by mature parr or anadromous male (Fig. 3, compare MM with MP). However, offspring from F3xMP3 grew faster than offspring from F3xMM3 in the downstream reach (Fig. 3). The same trend was observed, although not significantly, for males originating from these crosses in the upstream reach (Fig. 3).

### Incidence of early male maturation

Incidence of early male maturation showed a high variation between crosses and habitats ranging from 12 to 81% upstream (Fig. 2a) and from 10 to 93% downstream (Fig. 2b). Incidence of early maturation among males was explained by spring weight and cross but not by habitat as main effect (Table 1). Significant interacting effects were found for spring weight and cross, spring weight and habitat and the triple interaction between spring weight, cross and habitat (Table 1). Multiple comparison of the means showed that the incidence of early male maturation differs between maternal half-sib families (Fig. 2) with males from the F3xMP3 array (mature parr sire) showing a higher incidence of early male maturation (81 and 93%) than males from the F3xMM3 array (anadromous sire, 12 and 10%) independently of the channel reach. The same trend, although not significant, was observed for the F2xMP4 array with an incidence of early male maturation of 75 and 92%, compared to the F2xMM2 array which showed an early male maturation incidence of 27 and 64%.

### Early male maturation heritability and maturation reaction norm estimation

Individual maturation threshold values estimated by the LETM analysis varied greatly between crosses but not between habitats (Fig. 4). Accordingly, a high proportion of the phenotypic variance was due to the genetic variance which translated into a high early male maturation heritability H^2^ of 0.90 [0.67–1.00] (posterior median and 95% high probability density interval). Within family, maturation threshold estimates in the two reaches were similar (Fig. 4). Small difference in spring weight (observable cue) between reaches did not translate into different maturation reaction norm shapes but instead in the location along the maturation reaction norms explaining differences in early mature parr incidence between reaches (Fig. 4). While the different families showed similar spring weight distribution, large differences in maturation threshold alone (Fig. 4) explained large differences in incidence of early male maturation observed between families (Fig. 2). In our experiment, the maturation threshold varied depending on the male used for the cross within female (comparing Fig. 4a and c and Fig. 4b and d) illustrating the strong genetic effect on the early maturation strategy. Offspring sired by an early mature male (Fig. 4c and d) showed a much lower maturation threshold estimate when compared to offspring sired by an anadromous male (Fig. 4a and b respectively) all other factors (crossing female and rearing environment) being equal.

### Linkage mapping and QTL detection

From the Genotyping By Sequencing (GBS) approach, a total of 8274 diallelic SNPs were identified following the GATK Best Practices protocol among the three parents and 142 offspring that were successfully genotyped (75 in the F2xMM2 family and 67 in the F2xMP4 family). Further stringent quality filtering of the data removing (i) highly heterogyzote SNPs with inbreeding coefficient lower than −0.90 corresponding to paralogs, (ii) individual genotypes with a Genotype Quality (GQ) score lower than 10 and (iii) SNPs genotyped in less than 70% of the individuals resulted in a final set of 1367 high quality SNPs. A SNP typing error rate of 0.81% (104 discordant genotypes out of 12869 compared genotypes) was estimated from repeated typing of ten individuals. The additional comparison of four of the SNPs genotyped again in all individuals using an Agena Bioscience MassArray system identified no mismatch between the GBS SNPs data and the array SNPs data (no discordance out of 1192 compared genotypes).

A total of 51 microsatellites, 29 insertion-deletions, 24 SNP from the Agena Bioscience MassArray and 1134 SNPs from the GBS were informative in at least one family and used to construct the linkage map. The linkage map comprises 29 linkage groups (LG), each corresponding to one chromosome in the reference genome. LG was numbered according to its corresponding chromosome number in the reference genome for clarity. The linkage map presents a total of 484 non redundant positions and has a total size of 701 cM for the male map and 2009 cM for the female map (Supplementary Material File 3).

For the maturation threshold, the QTL model explains 20.6% of the phenotypic variance (Table 2). The model identified only one genome wide significant QTL for the maturation threshold in the linkage group 25 (Fig. 5a). No significant QTL at the genome level for maturation status or spring weight was identified. However four additional significant QTLs at the chromosome level were identified (Table 2) for maturation status in LG 14 (Fig. 5b, percentage of explained variance (PEV): 13.2%), LG 19 (Fig. 5c, PEV: 15.1%) and LG25 (Fig. 5d, PEV: 16.3%) and for spring weight in LG19 (Fig. 5c, PEV: 13.2%). In LG19, the QTL for maturation status collocated with the QTL for spring weight indicating that in this genomic region, genetic effect increasing early growth trigger sexual maturation in males. This QTL further showed high additive and dominance allelic effects for male MM2 and cross F2xMM2 (Table 2). In LG25 and LG14, maturation status QTL did not collocated with QTL for spring weight. Instead, the QTL for maturation status in LG 25 collocated with the highly significant QTL for maturation threshold indicting that this genomic region harbours genetic effects linked to maturation thresholds. Additive allelic effects on the LG25 QTLs showed similar estimates across parents but with higher value for the maturation threshold than for the maturation status (Table 2) while dominance effects on these QTLs were higher for the F2xMP4 cross compared to the F2xMM2 one. The estimated location of LG25 maturation threshold QTL spanned a large region including 29 positioned markers representing 6 non redundant genomic position (Fig. 6). Notably, candidate SNPs located near the Vgll3 gene (sqSNP_22 and sqSNP_24) and within the Akap11 gene (sqSNP_25), previously shown to be linked to sea age at maturation for anadromous Atlantic salmon^31^,^32^, fell within the interval of the QTL region but outside the QTL region with the highest statistical support (Fig. 6).

## Discussion

Alternative mating tactics have profound ecological and evolutionary implications. However they depend on complex genetic and environmental interactions that can be empirically difficult to decipher. According to the environmental threshold model, alternative phenotype expression depends on a normally distributed liability trait under environmental and polygenic control and a genetically determined threshold such as when the liability trait surpassed the threshold, the alternative phenotype is expressed^7^,^8^. Our study is a rare empirical application of the environmental threshold model (but see refs 12,13) which reports unequivocal evidence for a genetically determined threshold and further characterises the genetic architecture for the components of a threshold trait involved in alternative life histories in a vertebrate.

Indeed, variability in early male maturation among full-sibs families was high ranging from 11 to 86% and translated into a high heritability estimate of 0.90 [0.67–1.00]. Most notably, the difference in incidence of early male maturation found between sire phenotypes (84.6% versus 28.9% for offspring from mature parr and anadromous males respectively) is far more pronounced than results obtained from similar controlled crosses previously reported from artificial rearing in laboratory conditions (6.9% versus 0%^26^; 25% versus 15%^33^; 50.0% versus 31.9%^24^) highlighting the need for ecologically realistic experimental conditions for phenotypic expression research. Such contrasted findings can be due to the fact that, in the present experiment the maturation status of anadromous males was ascertained by selecting male sires that performed a seawater migration during their first year (scale inspection). Such a life history trait decreases drastically the risk of an early male maturation for these anadromous males used in crosses. Nonetheless, rearing fish in an ecologically realistic semi-natural environment is paramount to guarantee the expression of territoriality, which is a strong characteristic of salmon parr behaviour, as well as triggering physiological and developmental timelines as close as possible to wild characteristics thanks to natural temperature and feeding^34^. It is likely that the semi-natural rearing conditions used in this study created differences in growing patterns and selective mortalities than those experienced in laboratory or aquaculture environments. As such, the semi-natural rearing may have enlightened complex genetic and environmental interactions acting on early maturation. Indeed, effects of interactions were detected in liability trait distributions that differed between the two habitats: faster growth rate in the downstream reach (Fig. 3) generally translated into higher incidence of early male maturation (Fig. 2). However, very little (if any) environmentally-induced threshold variation was detected (Fig. 4). It should be noted that the protection from bird predation might have enhanced weak and small fish survival leading to a potential decrease of the mean growth rate compared to natural populations. However, such effect was probably marginal since fish size in October reached 76 mm on average which was within the range of wild young of the year salmon in France (71–97 mm^35^).

In the case of *S. salar*, previous studies reported substantial evidence for the role of genetics and environmental factors in controlling the liability trait distribution which in turn determines alternative mating tactics. Aubin-Horth & Dodson^36^ found in a natural population that future early male maturation at age one was found to be determined by early individual growth rate 20 days after hatching indicating either direct maternal effect or indirect parental genetic effect on early growth rate. Parental genetic effects and environmental (maternal) effects could not be distinguished due to the lack of pedigree information. However, when maternal effect is eliminated using controlled crosses, Garant *et al*.^22^ found a significant effect of paternal phenotype on offspring early growth rate showing that offspring from mature parr could quickly reach the maturation threshold and develop as mature parr themselves. Our results provide additional insights on the determinism of early male maturation in light of the environmental threshold model. First, the observable cue (spring growth) distribution did not differ between sire phenotypes after controlling for potential maternal and environmental effects. Second, the proportion of mature parr was much higher in offspring fathered by mature parr compared to offspring fathered by anadromous males that did not mature as parr, all other things being equal. Consistent replication of the results for two independent crosses and two environments indicates that a genetically determined threshold is a decisive factor in the occurrence of alternative mating phenotypes. Mean threshold values strongly differ between sire phenotype but threshold variance was lower for offspring from mature parr than for offspring from anadromous males. Within the framework of the environmental threshold model, this result suggests a bigger difference in fitness between mating tactics for offspring fathered by mature parr^7^. This hypothesis may further explained the dominance genetic effect detected for the maturation threshold QTL in LG25 that was observed for the family sired by a mature parr but not for the family sired by an anadromous male. Nevertheless, the identification of a QTL linked to maturation status in LG25 that collocated with a highly significant QTL for maturation threshold and the absence of QTLs for spring weight in the same location suggests the absence of a genetic correlation between early growth and maturation status at this particular genomic region, indicating that the two traits can evolve independently in response to selection, in support of the environmental threshold model^7^,^12^,^14^. Such configuration was not a general result however, as some genomic regions, such as LG19, showed collocation of QTLs for maturation status and spring weight indicating that genetic effects linked to spring weight (the observable cue, potentially resulting from some environmental effect) can also trigger early male maturation to some extent. The diverse genomics QTL configuration linked to the components of a threshold trait illustrated here, clearly shows the usefulness of a model such as LETM to isolate genetic from genetic-by-environment effect at the genome level.

A total of nine QTLs for early maturation have been previously reported^37^, but none of them collocated with the three QTLs detected here for early male parr maturation status. Differences between studies may be explained by the low number of families used preventing generalization of the results, differences in controlled-cross design and rearing environment conditions which may have led to contrasted phenotypic expression. Interestingly, the only significant QTL at the genome wide level detected for maturation threshold located in LG25 which includes the gene Vgll3 previously shown to contain genetic variation explaining up 39% of the variance in sea age at maturation for anadromous *S. salar*^31^,^32^ and to include a rare example of sex dependant dominant genetic effect^31^. The similarity between the current study and Barson *et al*. study^31^, in the location and dominance effect of the QTL for early male parr maturation threshold is intriguing but there is no evidence that Vgll3 gene is involved in early male parr maturation. Firstly, the QTL linked to maturation threshold covers a large genomic region probably including hundreds of genes. Secondly, the SNPs within Vgll3 are located in the vicinity of the QTL region far from the most statistically supported QTL location. Lastly, if sea age at maturation and male parr maturation could appear to be physiologically related traits, evolutionary constraints should greatly differ between the two traits. While age at maturation is a conflicting trait between sexes, resolved by sex dependence dominance^31^, male parr maturation is a conflicting trait between reproductive tactics within males, a different evolutionary conflict that may have led to a different genetic architecture. Therefore, it would be interesting to compare the evolutionary mechanisms that resulted in genetic effects on alternative mating tactics and resolution of their evolutionary constraints at the genomic level with those of such similar reproductive traits under contrasted evolutionary constrains. Further investigations would benefit from combining both quantitative genetics and population genomics approaches in the wild, using long term monitoring available for some *S. salar* populations, to refine the characterization of early male maturation complex genetic effects and genomic architecture and to estimate *in situ* alternative mating tactics fitness across generations^17^,^38^.

## Methods

### Ethical statement

This research followed European and national regulations and has been conducted after approval granted from the legal representative concerning fish capture and field work (prefectoral decrees n°2012230-0017 and n°2011-DDTM-SE-08) and from the ethical committee C2EA-73 *Comité d’Ethique Aquitaine Poisons Oiseaux* relative to the protocol implementation. All methods were performed following these relevant guidelines and regulations.

### Biological material and controlled-cross design

Given the constrains due to the rearing environment and the high number of individuals within family needed to estimate maturation reaction norms and to detect QTLs, the controlled-cross design was implemented to maximize maturation variability by including an anadromous males and a mature parr as sires crossed with the same female in order to rear 300 full-sibs per family. The crosses were replicated independently using a second triplet to control for any individual effects. Moreover, in order to ensure that males used in controlled crosses had contrasted phenotypes, scales were inspected under a stereomicroscope to check that anadromous males selected as fathers spent only one year in freshwater, thereby excluding the possibility that they had matured early in freshwater prior to seaward migration. In December 2011, two females (F2 and F3), two anadromous males (MM2 and MM3) and two mature male parrs of the year (MP3 and MP4) were caught in the Oir River (Normandie, France) and transported to nearby facilities (Moulin de Cerisel, Ducey, France) for gamete collection. Gametes were obtained from anesthetized individuals and transferred in temperature-controlled containers (2 to 4 °C) to the INRA ECOBIOP Fish Behavioural Ecology experimental facility in Ainhoa (Pyrénées Atlantique, France) where ova from each female were divided in two equal batches, each receiving sperm from one anadromous male or one mature parr resulting in four full-sib families nested into two maternal half-sib families (F2xMM2, F2xMP4, F3xMM3, F3xMP3). Eggs were incubated at 9–12 °C in the dark for 25 days, alevins were then exposed to ambient dim light photoperiod for 53 days until yolk sac absorption was complete.

### Experimental design and data acquisition

The number of individual reared per family (N = 300 × 4 full sib arrays) was determined to keep individual density within carrying capacity of the experimental channel (natural feeding from invertebrates stream production) but to have a sufficient number of male offspring at the end of the experiment for subsequent statistical analysis. In order to assess the effect of growth on the incidence of male parr maturation, the experimental channel (130 m long and 2.8 m wide) was divided into two reaches to run the experiment under two contrasted habitats: the upstream reach was exposed to shade from a large tree canopy most of the day and received a water flow of 220 m^3^.h^−1^; while the downstream reach was under direct sunlight with a lower water flow (about 180 m^3^.h^−1^). Higher growth was expected downstream with a higher amount of natural food available thanks to sunlight increasing biological productivity (primary producers and invertebrates) and a lower energy demand to swim in the water current.

In March 2012, 150 individuals per family were released in each reach corresponding to a starting fish density of 4 fish.m^−^^2^ which is comparable to early life stage fish density in natural habitats^39^. The entire channel was protected from bird predation using nets. In addition, each reach was delimited by four nets (two upstream and two downstream) in order to avoid any escape. Nets were connected to traps which allowed the cleaning of the nets with low disturbance as well as individual dispersal downstream to be checked daily. First time migrants were fin clipped and released upstream in their reach, while second time migrants were removed from the experiment to allow for natural fish density regulation.

**Figure 1:**
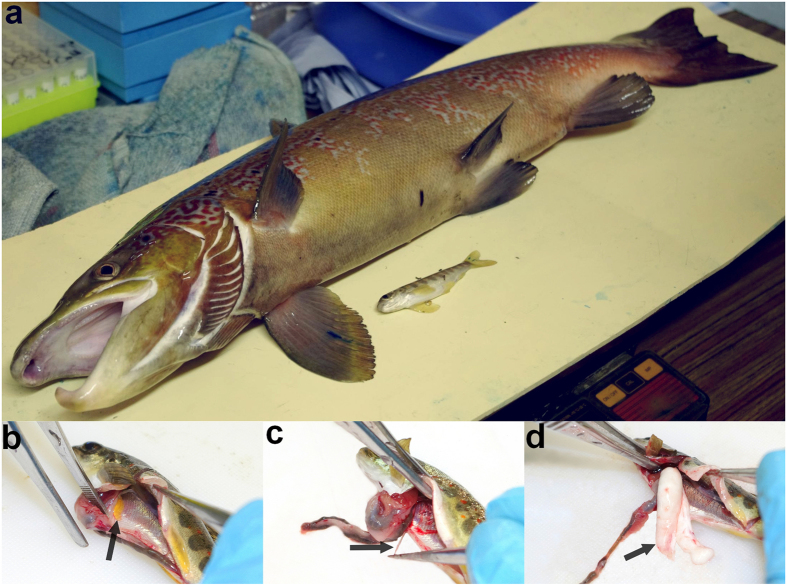
Illustration of the variability of male reproductive tactics in Atlantic salmon. (**a**) Sampling of two sexually mature males illustrating the variability of male reproductive tactics in Atlantic salmon. The anadromous male spent about 10 months in freshwater before migrating to the ocean and returning to freshwater a year later (length: 676 mm, weight: 2792 g). The early mature male parr is about 11 months old and only lived in freshwater (length: 86 mm, weight: 7 g). (b-d) Dissection of reared 10 months old individuals to record sex ((**b**) female and (**c,d**) males) and maturation status ((**c**) immature male parr, (**d**) mature male parr), with arrows indicating ovaries (**b**) and testis (**c,d**).

**Figure 2:**
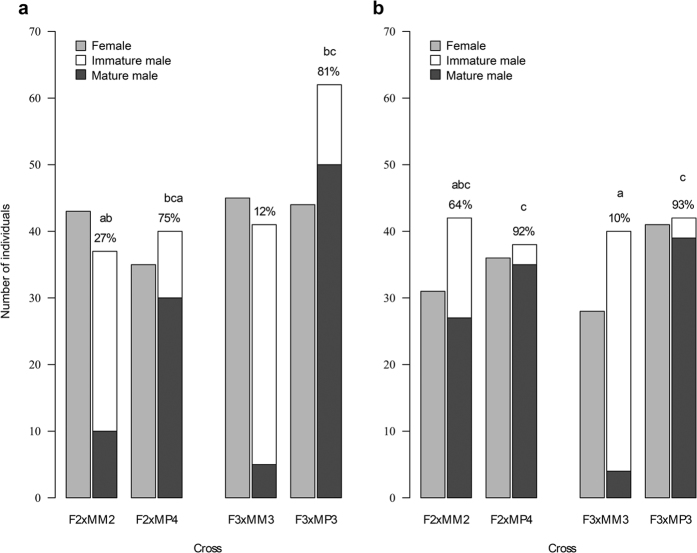
Number of females, immature and mature males among the full-sib families in the upstream (**a**) and downstream (**b**) reaches of the experimental channel. Percentage of male offspring that early mature are indicated; letters indicate a significant difference between proportion of early mature male across families and channel sections at a 0.05 probability level according to a Tukey multiple comparison test.

**Figure 3:**
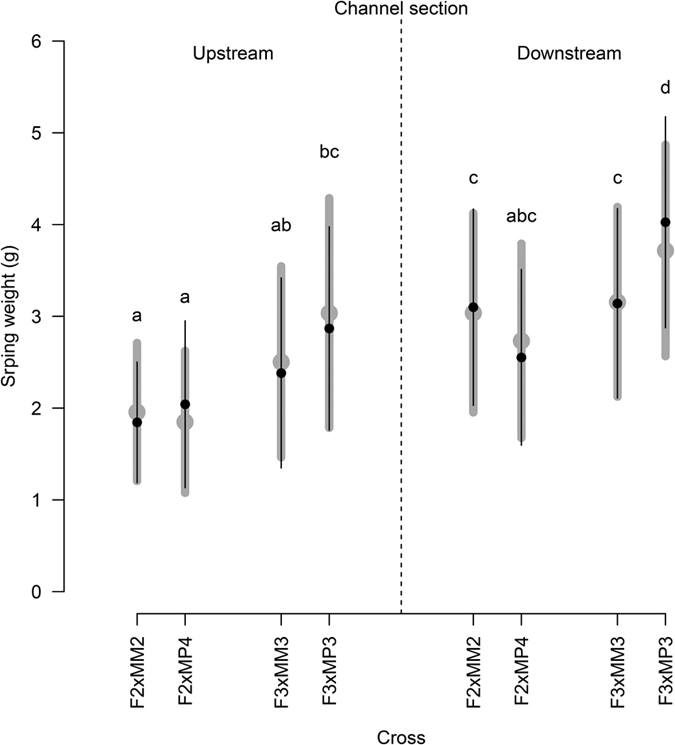
Spring weight of individuals for each family in the upstream (**a**) and downstream (**b**) channel reach. The mean (point) and standard deviation (line) are shown for all individuals in grey and for males only in black. Letters indicate a significant difference between spring weights of males at a 0.05 probability level according to a Tukey multiple comparison test.

**Figure 4:**
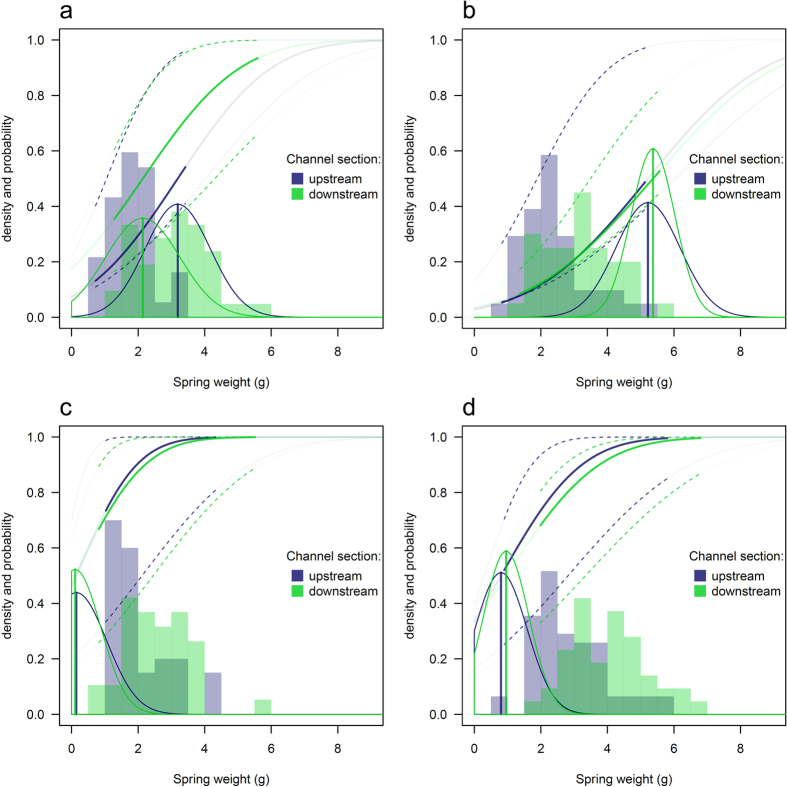
Distribution of spring weight (histograms), distribution of maturation threshold (bell shaped curves) and corresponding maturation reaction norm, i.e. the probability for a male parr to sexually mature early as a function of spring weigh (lines) estimated using LETM for the different crosses: (**a**) F2MM2, (**b**) F3MM3, (**c**) F2MP4 and (**d**) F3MP3. In each subplot, estimation from offspring reared in the upstream (dark blue) and downstream (light green) channel reach are shown. Histograms represent spring weight distribution (observable cue), vertical lines and bell-shaped curves show the median and distribution of estimated individual maturation thresholds, and solid lines represent the maturation reaction norm (dashed lines its 95% credibility interval) giving the probability for a male parr to sexually mature as a function of the observable cue computed from equation 7 in ref. 6.

**Figure 5:**
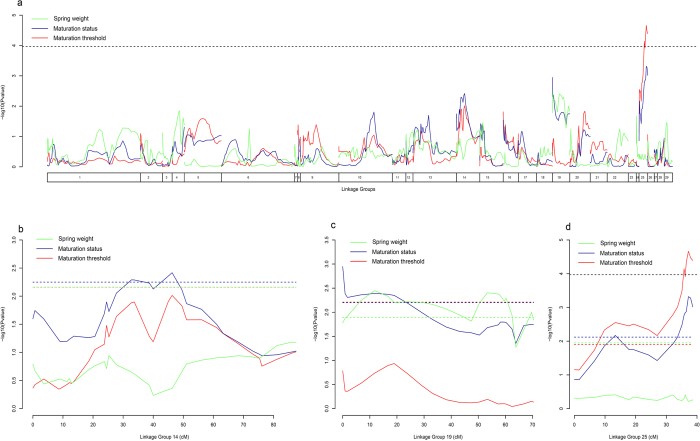
Results of the QTL detection for spring weight (green), maturation status (blue) and maturation threshold (red) across linkage groups (**a**) and for linkage groups LG14 (**b**), LG19 (**c**) and LG25 (**d**) where significant QTL were identified. X-axis indicates genomic position across linkage groups (**a**) and fine scale location within linkage group in centimorgan (**b,c** and **d**) and Y-axis gives the decimal logarithm of the Fisher test P value for the presence of a QTL. Continuous colored lines represent the log10 Fisher P value for the presence of a QTL at the specific location for each trait. Dashed black line represent the genome wide significance threshold for the log_10_ P value and colored dashed lines represent trait specific chromosome wide significance threshold for the three traits. Genome wide and chromosome wide significant QTL are located in the region where the log10 Fisher P value statistic test exceeds the significance threshold.

**Figure 6:**
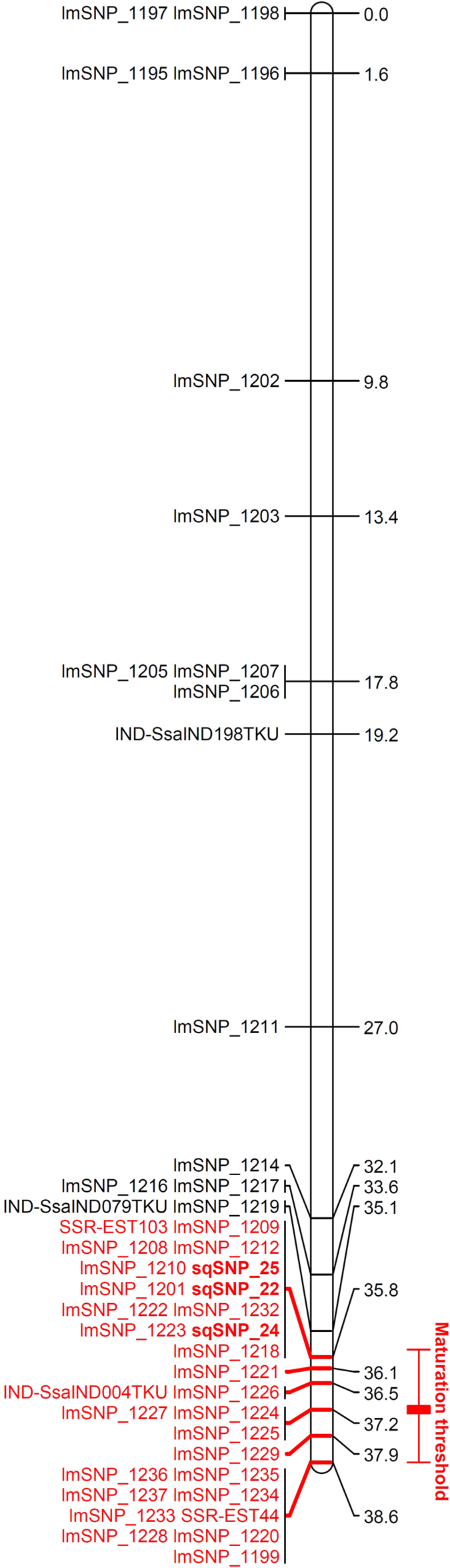
Maturation threshold QTL location on linkage group LG25. Marker locations are indicated in centimorgan, with in red the markers located within the QTL region. SNP sqSNP_22, sqSNP_24 and sqSNP_25, highlighted in bold, correspond to three SNP located on chromosome 25: respectively, a SNP without RefSeq reference located in position 28666061, rs863782897 located in position 28667099, both downstream Vgll3 gene, and rs863917458 located in position 28720779 within an intron of AKAP11 gene; these three markers were found, among others in the region, to be linked with sea age at maturation in anadromous Atlantic salmon^31^,^32^. These SNP were genotyped using a Sequenom array. lmSNP represents SNP genotyped using a Genotyping By Sequencing approach, markers beginning with IND are insertion-deletion and SSR identifies microsatellites.

In June 2012 individual survival and growth was monitored by electrofishing. Each individual was anesthetised, measured, weighted and fin clipped, then released after full recovery. The same procedure was repeated in October 2012 during which each individual was anesthetised measured, weighted, then killed by anaesthetics overdose, fin clipped and dissected to record sex and maturation status by visual inspection of the gonads (Fig. 1c,d).

### Individual genotyping and parentage assignment

Fin samples were used to genotype individuals at neutral nuclear markers first to identify parents of each individual and second to track individual records between the two electrofishing sessions. Such indirect identification from individual genotypes allowed us to relate growth rate with sex and maturity status without use of invasive tagging methods.

DNA extraction was performed using a NaCl/chloroform based protocol^40^. Previous protocols^41^,^42^,^43^ were optimised to amplify 15 microsatellite loci and one sex-specific locus^44^ in a single highly multiplexed PCR reaction using universal primers for fluorescent labelling^45^ to reduce genotyping time and cost (Supplementary Material File 1). PCR products were visualised as fluorescent profiles on an ABI 3100 Avant sequencer, scored manually using STRand^46^ and exported to MsatAllele package^47^ in R version 2.13.0 48 to bin raw allele sizes.

GenClone version 2.0 49 was used to match individual genotypes between the first (spring) and the final electrofishing session (fall). The analysis was performed independently for individuals in each channel reach. A pair of identical multilocus genotypes (or a pair of genotypes distant by only one allele across loci to allow for genotyping error) sampled in spring and fall was recorded as a valid recapture. Unique genotypes were recorded as a failure to capture individuals in any of the fishing session or as mortality during rearing. Subsequent analysis were performed solely on individuals captured both in spring and in fall in order to relate early growth rate in spring to male maturation in the fall^36^.

Colony version 2.0 50,51 was used to assign parentage using genitor genotypes as candidate parents of offspring sampled in the fall. Potential genotyping error was accounted for by setting a 1% allelic dropout rate (E1) and 1% of other kind of error (E2) as a prior for each locus. We used the Full Likelihood method with medium run length allowing for polygamy for both male and female. We ran the analysis twice using different random seed numbers to ascertain convergence of the analysis and stability of the results. The phenotypic and pedigree data are available in DRYAD (doi: 10.5061/dryad.4h0d0).

### Spring weight and incidence of early male maturation across channel reaches and families

Difference in individual growth rate across sexes, channel reaches and families was assessed by analysis of variance (ANOVA) in R version 2.13.0 48 with spring weight as response variable modelled by sex, cross, habitat and all two way interactions as explanatory factors. Difference in male growth rate between channel reaches and families was assessed using an ANOVA with spring weight modelled as a function of cross, habitat and interaction. Tukey honest significant differences was used to assess significance of mean spring weight difference between offspring from the different crosses reared in the two channel reaches while controlling for multiple tests.

The effect of spring weight, cross, habitat and all interactions on the incidence of early male maturation was tested using a generalised linear model with a binomial error distribution implemented in R version 2.13.0 48. Tukey honest significant differences was used to assess significance of early male maturation proportion between crosses reared in each channel reach while controlling for multiple tests.

### Maturation threshold and reaction norm estimation

In the Environmental Threshold model (ET), the individual dichotomous phenotype *Y*_*i*_ is determined by the comparison between a proximate cue *ƞ*_*i*_ with a threshold *θ*_*i*_:


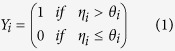


The ET model assumes that the triggering threshold is determined purely on a genetic basis, while the underlying proximate cue, the liability, is purely environmentally controlled^6^,^14^.

The Latent Environmental Threshold Model (LETM)^6^ is a recent extension of the ET model^9^ allowing to make statistical inference from empirical data. First, because the proximate cue is unknown and unobservable, the LETM treats the proximate cue as a latent factor that is imperfectly correlated with an observable cue. The distribution of the unknown proximate cue *ƞ*_*i*_ is expressed conditionally on the observable proxy *X*_*i*_ with some residual error given by *σ*_*η*_:


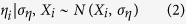


Second, the LETM uses the genetic relatedness among individuals to disentangle the sources of variation in the alternative phenotype, namely the threshold genetic variance and the liability environmental variance. The threshold *θ*_*i*_ varies among individuals and is a polygenic quantitative trait that is normally distributed, as typically assumed in quantitative genetics. Thus, the threshold *θ*_*i*_ is inferred from the mean threshold of the population *μ*_*θ*_ and the individual additive genetic effects *a*_*i*_:


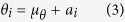


The additive genetic value *a* is assumed to covary according to the individual relatedness. Then, the additive genetic value of the individual i (*a*_*i*_) is normally distributed with the mean 0 and the additive genetic variation of the population 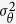 associated with the additive genetic relationship matrix A (containing all the pairwise values of relatedness between individuals):


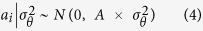


The LETM hence estimates the individual latent variables of primary interest, i.e., the proximate cue and the threshold trait, and allows comparing the distributions of threshold and cue between families and treatments (see refs 6 and 14 for more details on the model). Thus, the LETM was applied to estimate individual early male maturation threshold using individual maturation status (the observable alternative phenotype) and spring weight as an observable environmental cue *X*_*i*_ because early growth rate prior to maturation has been shown to influence the incidence of maturation^36^.

Model parameters were estimated using the whole data from the four families with a Bayesian approach implemented in the R package rjags^14^,^52^ (available at https://github.com/matbuoro/LETM) using two independent chains consisting of a burn-in of 5000 iterations followed by 20000 records every 100 iterations to estimate parameter posterior distribution. Median early parr maturation heritability and 95% credibility interval estimated by the model were reported. In addition, individual maturation thresholds (*θ*_*i*_) were estimated and their distributions were plotted for each cross and rearing channel reach to represent mean maturation threshold and maturation reaction norm corresponding to each experimental condition using equation (7) from^6^:


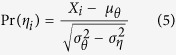


### Linkage mapping and quantitative trait locus (QTL) analysis

Linkage map construction and QTL detection was performed on a random sample of 78 young males from the F2xMM2 cross and 78 young males from the F2xMP4 cross selected for additional genotyping with their parents using a set of 77 microsatellite^53^ and 76 insertion-deletion markers^43^ has been genotyped as described above. In addition, a Genotyping By Sequencing (GBS) approach was applied^54^, sequencing 100 nucleotides upstream and downstream of each EcoT22 I restriction site to identify single nucleotide polymorphisms (SNP). The progeny arrays were sequenced in two lanes of an Illumina HiSeq 2000 sequencer (single end 100 bp) at the Genomic Diversity Facility of the Cornell University with 10 repeated individuals to assess the genotyping error rate. Raw reads were quality filtered using FASTX-Toolkit (Gordon A, Hannon GJ, unpublished), demultiplexed using Stacks component process_radtags^55^, adaptor trimmed using cutadapt software^56^. Resulting reads are deposited into the NCBI Short Read Archive (accession number SRP087385). Bowtie 2 57 was used to align reads to the Atlantic salmon reference genome assembly^58^ (GenBank: GCA_000233375.4) and GATK^59^ was used to identify and genotype SNP following GATK Best Practices recommendations^60^,^61^.

A set of 40 SNPs was selected for additional genotyping using an Agena Bioscience MassArray system (Supplementary Material File 2). Six SNPs linked to sea age at maturity in anadromous Atlantic salmon^31^, 6 SNPs obtained from the same GBS approach in natural populations (unpublished) and 24 SNPs identified in a RNAseq experiment (unpublished) were included in the genotyping array. In addition, four SNPs obtained from the GBS approach described above were regenotyped in all individuals to assess the accuracy of the GBS based SNP genotyping analysis. Genotype data is available in DRYAD (doi: 10.5061/dryad.4h0d0).

Linkage map construction was performed using Lep-MAP2^62^,^63^ accounting for sex-specific recombination rate because a lower recombination rate is established in male Atlantic salmon^64^,^65^. QTL detection for spring weight, maturation status and maturation threshold previously estimated using LETM was performed independently using phase determined by Lep-MAP2 and inter-markers distance of the female map with MCQTL Outbred module^66^,^67^ applying the iterative QTL detection method and the multipopulation connected model. This method assumes that the QTL locations are the same in all families, QTL genotypic effect are linked through multiple families’ relationship and four different alleles are assumed at the QTL. The -log_10_ Fisher test Pvalue thresholds corresponding to a type I error rate of 0.05 at the genome-wide level and at the chromosome level were determined using 1000 intra-family permutations of each trait data. The model assumes both additive and dominance genetic effects.

## Additional Information

**Accession codes:** NCBI Short Read Archive SRP087385, Dryad doi: 10.5061/dryad.4h0d0.

**How to cite this article:** Lepais, O. *et al*. Genetic architecture of threshold reaction norms for male alternative reproductive tactics in Atlantic salmon (*Salmo salar L.*). *Sci. Rep.* **7**, 43552; doi: 10.1038/srep43552 (2017).

**Publisher's note:** Springer Nature remains neutral with regard to jurisdictional claims in published maps and institutional affiliations.

## Supplementary Material

Supplementary Information

Supplementary Data 1

Supplementary Data 2

## Figures and Tables

**Table 1:** Analysis of deviance table for the generalized linear model testing the effect of spring weight, cross, reach and all interactions on the incidence of early male maturation.

Term	DF	Dev.	Res. DF	Res. dev.	P value
none			342	474.11	
SW	1	27.67	341	446.45	**<0.001**
CR	4	180.39	337	266.06	**<0.001**
CH	1	0.38	336	265.68	0.535
SW : CR	3	8.57	333	257.11	**0.036**
SW : CH	1	4.52	332	252.60	**0.034**
CR : CH	3	2.31	329	250.28	0.510
SW : CR : CH	3	13.91	326	236.37	**0.003**

SW: spring weight, CR: cross, CH: reach, DF: degree of freedom, Dev.: deviance, Res.: residual, P value: result from a chi-squared test comparing the reduction in deviance due to the added term to the residual.

**Table 2:** QTL model estimation for the dominance model.

Trait	Significance level	Linkage Group	QTL location (cM)^a^	−log(p.val)^b^	R^2^ (%)^c^	Additive effects^d^			Dominance effects^e^	
						F2	MM2	MP4	F2xMM2	F2xMP4
Maturation threshold	genome-wide	LG25	37.2 (35.6–38.6)	4.66	20.6	0.26	0.27	0.26	0.05	0.18
Spring weight	chromosome-wide	LG19	11.7 (0.0–70.4)	2.45	13.2	0.02	0.39	0.10	0.37	0.02
Maturation status	chromosome-wide	LG14	46.3 (0.5–57.2)	2.42	13.2	0.03	0.14	0.03	0.08	0.02
Maturation status	chromosome-wide	LG19	0.0 (0.0–22.6)	2.95	15.1	0.03	0.20	0.04	0.03	0.09
Maturation status	chromosome-wide	LG25	37.2 (35.4–38.6)	3.32	16.3	0.11	0.12	0.08	0.00	0.08

^a^QTL location with the strongest statistical support and range of the statistically supported QTL location in centimorgan;

^b^log_10_ of the P value for the Fisher test of significance of the QTL;

^c^proportion of phenotypic variance explained by the model;

^d^parent allelic additive effect in absolute value of the effect of one of the two QTL alleles;

^e^allelic dominance effect for parent pairs in absolute value of the effect of one of four QTL allele interactions.
