# Supplementary material for: Genetic architecture of threshold reaction norms for male alternative reproductive tactics in Atlantic salmon (Salmo salar L.)
Source: Sci Rep. 2017 Mar 10;7:43552. doi: 10.1038/srep43552 (PMC5345065; doi:10.1038/srep43552)
Supplement: Supplementary Information [file srep43552-s1.pdf]

Genetic architecture of threshold reaction norms for male alternative reproductive tactics in Atlantic salmon (*Salmo salar* L.). Lepais O, Manicki A, Glise S, Buoro M, Bardonnet A.

## Supplementary Information File

### Detailed protocol for multiplexed microsatellite genotyping

16 studied loci (Figure ESM1) consisted in SSsp2210, SSsp2215 and SSsp2216 (Patterson et al. 2004), Ssa85, Ssa197 and Ssa171 (O'Reilly et al. 1996), Ssa412UOS and Ssa407UOS (Cairney et al. 2000), SSOSL85 (Slettan et al. 1995), MHC1 (also known as Sasa-UBA, Grimholt et al. 2002),EST19, EST28, EST41, EST107 and EST123 (Vasemägi et al. 2005) and Yanng400, a male specific genetic marker (Yanng400F: AGACTGCTCATTGAAA and Yanng400R: TCAGGGAGAAGGGCTTGTAG; Guigen Y., personal communication) linked to the salmonids Y chromosome sequence (Yano et al. 2013). PCR amplification was carried out in a final volume of 5µL using 1X Type-it Microsatellite PCR Master Mix (Qiagen), unequal concentration of each of the unlabeled forward primer concatenated with an universal sequence (Blacket et al. 2012) and reverse primer for each loci (Integrated DNA Technology), the appropriated amount of each universal primer sequence (Blacket et al. 2012) labelled with 6-FAM, VIC, NED and PET fluorescent dye (Applied Biosystems) and about 20 ng of template DNA (i.e., 0.5µL of undiluted extracted DNA). PCR cycle were performed in a 2720 thermocycler (Applied Biosystems) consisting of a denaturing step of 5 min at 95°C, followed by 32 cycles of 95°C for 30 s, 59°C for 180 s, and 72°C for 30 s and a final elongation step of 30 min at 60°C. Amplified products were sized using a ABI 3100 Avant automated sequencer (Applied Biosystems) using GeneScan 500LIZ internal size standard (Life Technologies). Detailed lab bench recipe is provided in Tables ESM1 to ESM5 below.

**References:**


Blacket MJ, Robin C, Good RT, Lee SF, Millers AD (2012) Universal primers for fluorescent labelling of PCR fragments - an efficient and cost-effective approach to genotyping by fluorescence. *Molecular Ecology Resources* **12**:456-463.

Cairney M, Taggart JB, Høyheim B 2000. Characterization of microsatellite and minisatellite loci in Atlantic salmon (*Salmo salar* L.) and cross-species amplification in other salmonids. *Molecular Ecology* **9**:2175-2178.

Grimholt U, Drabløs F, Jørgensen SM, Høyheim B, Stet RJM (2002) The major histocompatibility class I locus in Atlantic salmon (Salmo salar L.): polymorphism, linkage analysis and protein modelling. *Immunogenetics* **54**:570-581.

O'Reilly PT, Hamilton LC, McConnell SK, Wright JM 1996. Rapid analysis of genetic variation in Atlantic salmon (*Salmo salar*) by PCR multiplexing of dinucleotide and tetranucleotide microsatellites. *Canadian Journal of Fisheries and Aquatic Sciences* **53**:2292-298.

Paterson S, Piertney SB, Knox D, Gilbey J, Verspoor E 2004. Characterization and PCR multiplexing of novel highly variable tetranucleotide Atlantic salmon (*Salmo salar* L.) microsatellites. *Molecular Ecology Notes* **4**:160-162.

Slettan A, Olsaker I, Lie O (1996) Polymorphic Atlantic salmon, *Salmo salar* L., microsatellites at the SSOSL438, SSOSL439 and SSOSL444 loci. Animal Genetics, 27, 57-58.

Vasemägi A, Nilsson J, Primmer CR (2005) Seventy-five EST-linked Atlantic salmon (*Salmo salar* L.) microsatellite markers and their cross-amplification in five salmonid species. Molecular Ecology Notes, 5, 282-288.

Yano A, Nicol B, Jouanno E, Quillet E, Fostier A, Guyomard R, Guiguen Y. The sexually dimorphic on the Y-chromosome gene (sdY) is a conserved male-specific Y-chromosome sequence in many salmonids. Evolutionary Applications, 6, 486-496.

Figure ESM1: Example of electrophoregram profile obtained from one individual (top panel) and corresponding allele range and fluorescent dye for each loci (bottom panel).

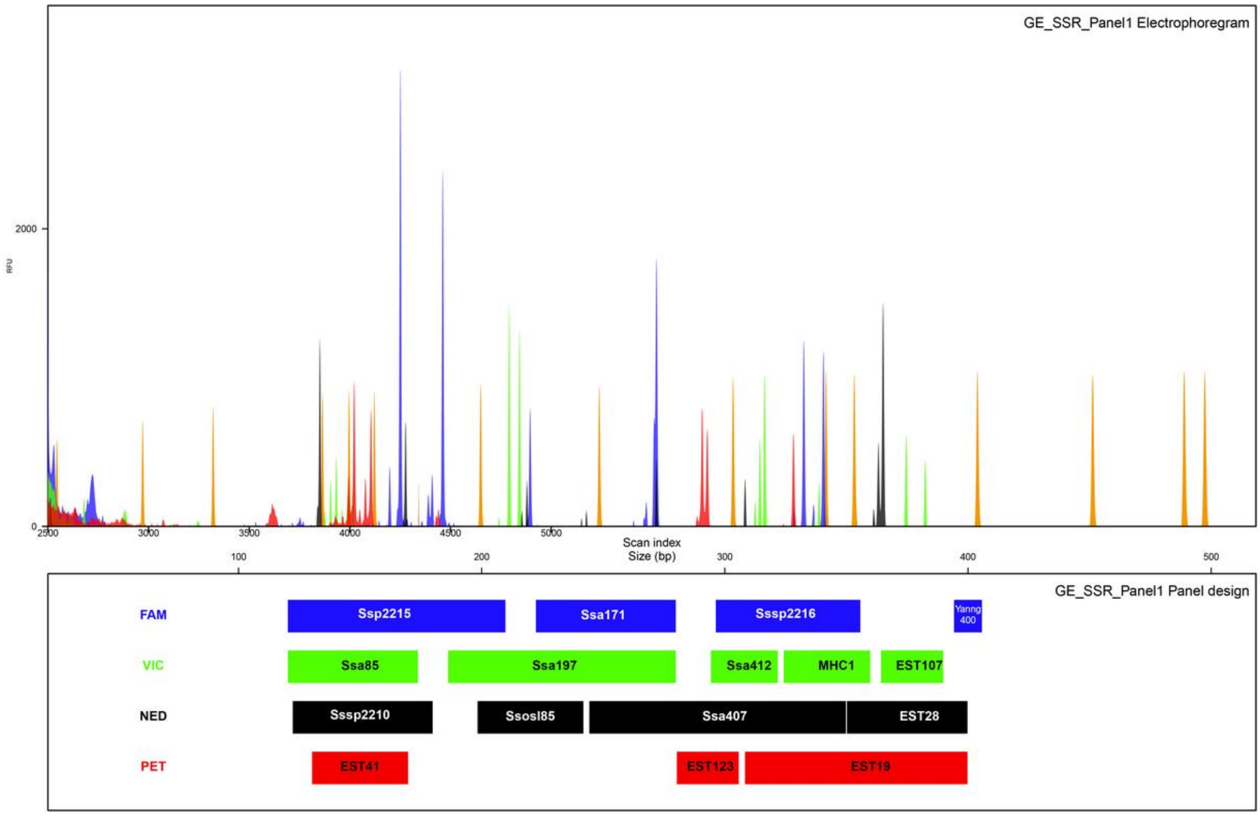

Table ESM1: Unlabelled primer sequences. Primer manes are omposed by concatenation of the loci name followed by the code of the universal sequence tag (A, B, C or D) that will match one ot the four labelled universal primer and finally the indication for reverse (R) or forward (F) primer.

| Unlabelled primer name | Sequence |
|---|---|
| Ssa171_A-F | GCCTTGCCAGCCCGCTCAGGAGGTCGCTGGGGTTTACTAT |
| Sssp2216_A-F | GCCTTGCCAGCCCGCTCAGCTCCTCCTGGGATTTCCTGTCA |
| Sssp2215_A-F | GCCTTGCCAGCCCGCTCAGGGTCAGTCAGTCACACCATGC |
| Ssa197_B-F | GCCTCCCTCGCGCCATGGCAGGGATTTGACATAAC |
| Ssa412_B-F | GCCTCCCTCGCGCCAGTGGAGATACACAGCACTTA |
| MHCI_B-F | GCCTCCCTCGCGCCAGGAGAGCTGCCCAGATGACTT |
| EST107_B-F | GCCTCCCTCGCGCCAAGCGTTTACGTCGAATCCAA |
| Ssa85_B-F | GCCTCCCTCGCGCCAACCCGCTCCTCACTTAATC |
| Ssa407_C-F | CAGGACCAGGCTACCGTGTCGTGACTACTAAGTCTTTGACCA |
| Ssosl85_C-F | CAGGACCAGGCTACCGTGTGTGGATTTTTGTATTATGTTA |
| EST28_C-F | CAGGACCAGGCTACCGTGCACAGGCACACACTCCTCAT |
| Sssp2210_C-F | CAGGACCAGGCTACCGTGCCTTTTTCCAATGGGATTCA |
| EST19_D-F | CGGAGAGCCGAGAGGTGCGCTTCCTGGACAAAAATTA |
| EST41_D-F | CGGAGAGCCGAGAGGTGTGCAAGTAAAGGCAGGGTTT |
| EST123_D-F | CGGAGAGCCGAGAGGTGGCGGCCCTTAGTGTAATCAA |
| Ssa171-R | TTATTATCCAAAGGGGTCAAAA |
| Sssp2216-R | GTTTCTGGAGCAGAGGATTGCTG |
| Sssp2215-R | GTTTGTCACTAGCCAGGTGTCC |
| Ssa197-R | GTTTGGGTTGAGTAGGGAGGCTTG |
| Ssa412-R | GTTTCTTGGTTAGTACCGGACATG |
| MHCI-R | GTTTCAATTACCACAAGCCCGCTC |
| EST107-R | GTTTCTCATGGAGGGTGGAAGTGT |
| Ssa85-R | GTTTCAAGCTACCCCATGCAGAG |
| Ssa407-R | GTTTGTGTAGGCAGGTGTGGAC |
| Ssosl85-R | GTTTATACATTTCCTCCTCATTCAG |
| EST28-R | GTTTCAGGTGAAGAGCATGACCAA |
| Sssp2210-R | GTTTCATGCACACACATTCACTGC |
| EST19-R | GTTTGAGCACACCCATTCTCA |
| EST41-R | GTTTGTGGTAGGATTGGGGTTCCT |
| EST123-R | GTTTCTCGCCAGTCACTCTTCAA |

Table ESM2: Fluorescent labelled universal primer sequence. Primer name are composed by the concatenation of the code of the universal sequence tag (A to D) followed by the fluoresnet dye used to label the primer.

| Labelled Primer name | Dye (5') | Sequence |
|---|---|---|
| A-FAM | FAM | GCCTTGCCAGCCCGCTCAG |
| B-VIC | VIC | GCCTCCCTCGCGCCA |
| C-NED | NED | CAGGACCAGGCTACCGTG |
| D-PET | PET | CGGAGAGCCGAGAGGTG |

Table ESM3: Primer volume for the 10X primer mixture.

| **10X Primer Mix recipe for a total of 300 µL (allowing for 5 96-well PCR plates)** | | |
|---|---|---|
| TE buffer (µL) | 82.8 | |
| Loci (from stock primer solution @ 100µM) | R (µL) | F (µL) |
| EST19 | 7.5 | 3.75 |
| Ssa407 | 7.5 | 3.75 |
| Ssosl85 | 7.5 | 3.75 |
| EST28 | 1.5 | 0.75 |
| Ssa197 | 3 | 1.5 |
| Ssa171 | 1.5 | 0.75 |
| Ssa412 | 4.5 | 2.25 |
| EST41 | 3 | 1.5 |
| MHCI | 15 | 7.5 |
| Sssp 2216 | 4.5 | 2.25 |
| EST123 | 7.5 | 3.75 |
| Sssp2215 | 0.6 | 0.3 |
| EST107 | 3 | 1.5 |
| Sssp2210 | 4.5 | 2.25 |
| Ssa85 | 22.5 | 11.25 |
| Yanng400 | 15 | 7.5 |
| A-FAM (µL) | 10.8 | |
| B-VIC (µL) | 24 | |
| C-NED (µL) | 10.5 | |
| D-PET (µL) | 9 | |

Table ESM4: Product volumes for the PCR mixture.

| **PCR Mix pour 110 individuals (a full 96-well PCR plate) with a final PCR volume of 5µL** | | | |
|---|---|---|---|
| * Qiagen 2X Multiplex Mix | 275 | µL | |
| 10X Primer Mix | 55 | µL | |
| * H2O µQ | 165 | µL | |
| total | 495 | → 61µL are putted in the wells of the first plate column and then 4.5µL are dispatch using a 8-multichannel pipette from the column 1 into columns numbers 2 to 12. This allow for about twice the volume in the first column (final PCR volume in the first column at 10µL) allowing to run 5µL of these PCR products in a 3% agarose gel (migration for 15 minutes at 400 v) to check for successful amplification. | |
| +ADN | 0.5 | µL | excepted for column 1 where 1µL of DAN should be addeed. |

* from the Qiagen Tuype-It Microsatellite PCR kit

Table ESM5: Parameter of the thermal cycler.

| **PCR cycles** | | |
|---|---|---|
| | 95°C | 5 min |
| X32 | 95°C | 30 s |
| | 59°C | **180 s** |
| | 72°C | 30 s |
| | 60°C | 30 min |
